# Downregulation of the tumor suppressor HSPB7, involved in the p53 pathway, in renal cell carcinoma by hypermethylation

**DOI:** 10.3892/ijo.2014.2314

**Published:** 2014-02-27

**Authors:** JIAYING LIN, ZHENZHONG DENG, CHIZU TANIKAWA, TARO SHUIN, TSUNEHARU MIKI, KOICHI MATSUDA, YUSUKE NAKAMURA

**Affiliations:** 1 Laboratory of Molecular Medicine, Human Genome Center, Institute of Medical Science, University of Tokyo, Tokyo 108-8639, Japan;; 2 Section of Hematology/Oncology, Department of Medicine, University of Chicago, Chicago, IL 60637, USA;; 3 Department of Urology, School of Medicine, Kochi University, Kochi 783-8505;; 4 Department of Urology, Kyoto Prefectural University of Medicine, Kyoto 602-8566, Japan

**Keywords:** HSPB7, renal cell carcinoma, hypermethylation, p53

## Abstract

In order to identify genes involved in renal carcinogenesis, we analyzed the expression profile of renal cell carcinomas (RCCs) using microarrays consisting of 27,648 cDNA or ESTs, and found a small heat shock protein, HSPB7, to be significantly and commonly downregulated in RCC. Subsequent quantitative PCR (qPCR) and immunohistochemical (IHC) analyses confirmed the downregulation of HSPB7 in RCC tissues and cancer cell lines in both transcriptional and protein levels. Bisulfite sequencing of a genomic region of HSPB7 detected DNA hypermethylation of some segments of HSPB7 in RCC cells and concordantly 5-aza-2′-deoxycytidine (5-Aza-dC) treatment of cancer cells restored HSPB7 expression significantly. Ectopic introduction of HSPB7 in five RCC cell lines remarkably suppressed cancer cell growth. Interestingly, we found that HSPB7 expression could be induced by p53 in a dose-dependent manner, indicating that this gene functions in the p53 pathway. Our results imply that HSBP7 is likely to be a tumor suppressor gene regulated by p53 and its downregulation by hypermethylation may play a critical role in renal carcinogenesis.

## Introduction

Renal cell carcinoma (RCC) accounts for approximately 2% of all cancers worldwide (1) and its incidence has increased by 2–3% in the last decade with even higher rate in developed countries (2–6). The underlying mechanisms such as some environmental and genetic risk factors including smoking, obesity, acquired cystic kidney disease and inherited susceptibility (von Hippel-Lindau disease) (3,7,8) have been indicated, but the etiological and pathological mechanisms of this disease are still far from fully understood.

Although local renal tumors can be surgically removed (9–11), distant metastasis is often observed even if the primary tumor is relatively small (12,13). Patients with metastatic RCC generally result in extremely poor outcomes with overall median survival of around 13 months and the 5 year survival rate of <10% (13). For the advanced-stage patients, systemic therapy including immunotherapy (e.g. IL-2, IFN-α) and/or molecular-targeted drugs (e.g. sunitinib, bevacizumab, sorafenib, temsirolimus and everolimus) is recommended (14), but the response rates are not satisfactory.

To better understand the molecular mechanisms of renal carcinogenesis and apply the information for the development of effective treatment and early diagnosis, we performed genome-wide gene expression profile analysis and identified a small heat shock protein, HSPB7, whose function in cancer is unknown, to be downregulated in a great majority of human RCC samples.

In this study, we attempted to address two key questions, i) whether HSPB7 has growth suppressive function and ii) how HSPB7 is downregulated in RCCs. We here report for the first time that HSPB7 is likely to be a tumor suppressor which is frequently downregulated by DNA methylation in RCCs and is involved in the p53 pathway.

## Materials and methods

### Tissue samples and cell lines

Tissue samples used in this study were obtained from patients with written informed consent at three hospitals: Juntendo University School of Medicine, Kochi University School of Medicine, and Kyoto Prefectural University of Medicine. The human RCC cell lines (Caki-1, Caki-2, 786-O, A-498, ACHN), HEK293 and NCI-H1299 (lung carcinoma, p53-null) were purchased from American Type Culture Collection (ATCC; Rockville, MD, USA). Colon cancer cell lines HCT116 p53 wild-type (p53^+/+^) and its derivative (p53^−/−^) were gifts from Dr B. Vogelstein (Johns-Hopkins University, Baltimore, MD, USA). Normal human renal proximal tubule epithelial cells (RPTEC) were purchased from Lonza Walkersville Inc. (Walkersville, MD, USA). All cell lines were grown in monolayers in appropriate media recommended by suppliers: Dulbecco’s modified Eagle’s medium (Gibco, Carlsbad, CA, USA) for HEK293, HCT116 (p53^−/−^) and HCT116 (p53^+/+^); Eagle’s minimal essential medium (Gibco) for A-498; McCoy’s 5A medium (Gibco) for Caki-1 and Caki-2; RPMI-1640 medium (Gibco) for ACHN, 786-O and NCT-H1299; in addition, cells were supplemented with 10% fetal bovine serum (Cell Culture Bioscience, Nichirei Biosciences, Inc., Tokyo, Japan) except ACHN (5%), and 1% penicillin-streptomycin-amphotericin B suspension (Wako, Osaka, Japan). RPTEC were grown in REGM™ BulletKit, purchased from Lonza Walkersville Inc. (Walkersville, MD, USA). All cells were maintained at 37°C in humid air with 5% CO_2_ condition. Cells were transfected with plasmids using FuGENE 6 transfection reagent (Roche, Basel, Switzerland) or Lipofectamine LTX and Plus reagent (Invitrogen, Carlsbad, CA, USA) according to the manufacturer’s protocols.

### cDNA microarray and selection of candidate genes

We prepared a genome-wide cDNA microarray with totally 27,648 cDNAs/ESTs selected from the UniGene database of the National Center for Biotechnology Information (NCBI). This microarray system was constructed as previously described (15,16). We analyzed 15 clear cell renal cell carcinomas (RCC) and selected candidate genes according to the following criteria: i) genes for which we were able to obtain expression data in more than 50% of the cancers examined; ii) genes whose expression ratio was <0.2 in more than 50% of informative cases; and iii) the function of the gene was still unknown. Through these criteria, several candidates including HSPB7 were further validated. Gene expression data were deposited in the Gene Expression Omnibus database (accession no. GSE39364).

### Quantitative real-time PCR (qPCR)

We extracted total RNA from the microdissected RCC clinical samples, microdissected normal renal cortex, 25 different normal organs (17) and cultured cells using RNeasy mini kits (Qiagen, Valencia, CA, USA). RNAs from cell lines were reversely transcribed using the oligo (dT)21 primer and SuperScript III reverse transcriptase (Invitrogen). RNAs from tissue samples were treated with DNase I and subjected to two rounds of RNA amplification using T7-based *in vitro* transcription (Epicentre Technologies, Madison, WI, USA), then amplified RNAs were reversely transcribed to single-stranded cDNAs using random primer with Superscript II reverse transcriptase (Invitrogen) according to the manufacturer’s instruction. qPCR was conducted using the SYBR-Green I Master (Roche) on a LightCycler 480 (Roche). Standard curve method was used for quantification analysis, and β2 micro-globulin (B2M) served as a control gene. The qPCR primers for HSPB7 in cell lines were: 5′-ACTTCTCACCTGAAGA CATCATTG-3′ (forward) and 5′-CATGACAGTGCCG TCAGC-3′ (reverse). The qPCR primers for HSPB7 in tissues were: 5′-GACCTTCCATCAGCCTTAACC-3′ (forward) and 5′-ATGTGGGAGACGAAACCAAG-3′ (reverse). The qPCR process was started at 95°C for 5 min, then underwent 45 cycles at 95°C for 10 sec, 55°C for 10 sec and 72°C for 10 sec. Data analysis including standard curve generation and copy number calculation was performed automatically. Each reaction was performed in duplicate and negative controls were included in each experiment.

### Immunohistochemistry (IHC)

A kidney tissue array (BioChain Institute, Inc., USA) was used to analyze the protein expression of HSPB7 by IHC staining. This tissue array included 11 cases of RCC with corresponding normal tissues from the same patients as controls. Tissue sections were deparaffinized, rehydrated, and processed under high pressure (125°C, 30 sec) in antigen-retrieval solution of pH 9.0 (S2367, Dako, Carpinteria, CA, USA). Sections were blocked with Protein Block Serum Free (Dako) for 1 h at room temperature, followed by incubation with primary antibody (HSPB7, 1:100, Proteintech, Chicago, IL, USA) overnight at 4°C. At day 2, endogenous peroxidase activity was blocked by incubation in 3% hydrogen peroxide for 30 min at room temperature. Sections were incubated with a secondary antibody (Dako Envision^+^ system-HRP labeled polymer anti-rabbit K4003) for 30 min at room temperature, followed by DAB staining (K3468, Dako), counter stained with hematoxylin QS (H-3404, Vector Laboratories, Burlingame, CA, USA), dehydrated and mounted. Three independent investigators semi-quantitatively assessed the HSPB7 positivity without prior knowledge of clinicopathological data. According to the intensity of HSPB7 staining, these samples were evaluated as: negative (−), weakly positive (+), moderate positive (++), and strong positive (+++). HSPB7 negative or weakly positive (−/+) were considered low expression, and moderate or strong positive were considered high expression (++/+++).

### 5-Aza-2′-deoxycytidine (5-Aza-dC) treatment

5-Aza-dC (Sigma-Aldrich, St. Louis, MO, USA) was dissolved in DMSO. For a negative control, 5 RCC cell lines were treated with DMSO alone for 4 days. For a 5-Aza-dC group, cells were treated with DMSO for 1 day, following 5-Aza-dC-treatment (1, 3 and 10 *μ*M, respectively) for 3 days. On the fifth day, total RNAs of all cells were isolated using the RNeasy mini kits (Qiagen, Valencia, CA, USA), according to the manufacturer’s directions. qPCR was subsequently performed to detect the expression of HSPB7. To detect the protein level of HSPB7 in 5 RCC cell lines after the same treatment (5-Aza-dC 1 *μ*M was used in 5-Aza-dC group), western blot and immunocyto-chemical (ICC) analyses were performed.

### Bisulfite sequencing

Genomic DNA was extracted from RPTEC, HEK293 and 5 RCC cell lines (Caki-1, Caki-2, 786-O, A-498, ACHN) using the DNeasy blood and tissue kit (Qiagen). Genomic DNA (3.5 *μ*g each) were digested at 37°C for 16 h with 35 units of *Xho*I (Takara, Tokyo, Japan) and 1X H buffer (Takara) in 50 *μ*l of reaction volume. After treatment with phenol/chloroform/isoamyl alcohol (25:24:1, v/v), the DNA was finally dissolved in TE buffer and denatured in 0.3 N NaOH for 20 min at 37°C, and then the unmethylated cytosine residues were sulfonated by incubation in 3.12 M of sodium bisulfite (pH 5.0, Sigma-Aldrich) and 0.5 mM of hydroquinone (Sigma-Aldrich) at 55°C for 16 h. The sulfonated DNA was recovered using the Nucleospin Extract II (Macherey-Nagel GmbH and Co. KG, Düren, Germany) according to the manufacturer’s recommendations. The conversion reaction was completed by desulfonating in 0.3 N NaOH for 20 min at 37°C. The DNA was ethanol precipitated, then washed by 70% ethanol and resuspended in TE buffer. Primers for bisulfite genomic sequencing PCR were designed by the use of the online program MethPrimer. The primers for region 1 were: 5′-TTT GAAGGGTTTTGGGTTTAATATAT-3′ (forward) and 5′-CTCCTAACTACAAACTATCCAACAC-3′ (reverse). The primers for region 2 were: 5′-GGGTTGGTTTTAAGTTT AGGGATAG-3′ (forward) and 5′-AAAAAAAATTCTA TAACTCATCCAC-3′ (reverse). The primers for region 3 were: 5′-TGTATAT TGATG GAG GAG GTATAGT-3′ (forward) and 5′-AAAAAAAACTAAAAATCTTCTCCC-3′ (reverse). The primers for region 4 were: 5′-TGGAGAAGG TTTTGAGTATGTTTTT-3′ (forward) and 5′-CCACAT CTATCCCTATAACCACATC-3′ (reverse). The amplification products were checked by electrophoresis. After gel purification, the PCR products were cloned into pCR2.1-TOPO vector (Invitrogen), and 10 or more colonies were randomly chosen and sequenced. Methylation level analysis was performed by using QUMA software (http://quma.cdb.riken.jp/).

### Construction of HSPB7 expression vector

To construct an HSPB7 expression vector, the entire coding sequence of HSPB7 cDNA (based on NM_014424.4 in Pubmed) was amplified by PCR using KOD-Plus DNA polymerase (Toyobo, Osaka, Japan). The primers used for PCR reaction were 5′-AAAGAATTCCGTCCGTGGATGAGCCACAG-3′ (forward) and 5′-TTTCTCGAGGATTTTGATCTCCGTC CGGA-3′ (reverse). The PCR product was inserted into the *Eco*RI (Takara) and *Xho*I (Takara) sites of pCAGGSnHC expression vector containing the HA tag. The sequence and protein expression for pCAGGSnHC-HSPB7-HA were confirmed by DNA sequencing, western blot and ICC analyses.

### Western blot analysis

To prepare whole cell extracts, cells were collected and lysed in chilled radioimmunoprecipitation assay buffer (RIPA) (50 mM Tris-HCl at pH 8.0, 150 mM sodium chloride, 0.1% SDS, 0.5% DOC, 1% NP-40), 1 mM phenyl methylsulphonyl fluoride (PMSF), 1 mM DTT and 0.1% Calbiochem Protease Inhibitor Cocktail Set III, EDTA-Free (EMD Chemicals Inc., Merck KGaA, Darmstadt, Germany). Following 15-min ultrasonication and subsequent 30-min incubation on ice, homogenates were centrifuged for 15 min at 4°C, and the supernatants were collected and boiled in SDS sample buffer. Each sample was loaded into a 15% SDS-polyacrylamide gel electrophoresis (SDS-PAGE) and transferred to a nitrocellulose membrane (Hybond^™^ ECL^™^, Amersham, Piscataway, NJ, USA). Protein bands on western blots were visualized by chemiluminescent detection (ECL, Amersham). The primary antibodies used in this study included rabbit anti-human HSPB7 polyclonal antibody (Proteintech, diluted 1:500) and goat anti-rabbit IgG-HRP secondary antibody (Santa Cruz Biotechnology, Santa Cruz, CA, USA, diluted 1:30,000).

### Immunocytochemistry (ICC)

Five RCC cell lines were seeded on Lab-Tek II chamber slide system (Nalge Nunc International). At day 5 after the 5-Aza-2′-dC-treatment, the cells were fixed with 4% paraformaldehyde in PBS for 10 min and permeabilized with 0.2% Triton X-100 in PBS for 5 min at room temperature. Cells were covered with blocking solution (3% BSA in PBS contained 0.2% Triton X-100) for 60 min at room temperature. Then the cells were incubated with rabbit anti-human HSPB7 polyclonal antibody (Proteintech, diluted 1:250) overnight at 4°C, following an Alexa Fluor 488 goat anti-rabbit IgG antibody (Molecular Probes, Eugene, OR, USA, diluted 1:1,000) for 1 h at room temperature. PBS or 0.2% Triton X-100 in PBS was used for washing after each step. Then cells were stained with DAPI (Vector) and viewed with a laser scanning spectral confocal microscope (Leica TCS SP2).

### Colony formation assay

Cells were plated in a 6-well plate and transfected with pCAGGSnHC-HSPB7-HA or empty vector using FuGENE 6 (ACHN and Caki-1) or lipofectamine LTX (Caki-2, A498 and 786-O) transfection reagent (Roche). After 48 h of transfection, cells were selected with G418 (Gibco) for 14–21 days. Colonies (>1 mm diameter) were counted using the Image J software after fixed with methanol and stained with 0.1% crystal violet. The experiment was carried out twice in duplicate wells.

### DNA-damaging treatments

When cells reached 60–70% confluence in the culture dish, HCT116 (p53^−/−^) and HCT116 (p53^+/+^) cells were incubated with adriamycin for 2 h at the indicated concentration. The cells were harvested at different time points after cell-damaging treatment as indicated in the figure legends. Replication-deficient recombinant adenovirus encoding p53 (Ad-p53) or LacZ (Ad-LacZ) was generated and purified as previously described (18,19). NCI-H1299 lung cancer cells were infected with viral solutions at an indicated multiplicity of infection (MOI) and incubated at 37°C until harvest.

### p53-binding site screening by Luciferase assay

Two DNA fragments including candidate p53-binding sites of HSPB7 were amplified by PCR, digested with *Mlu*I and *Bgl*II and cloned into pGL3-Promoter vector (Promega, Madison, WI, USA). Primer sequences (including *Mlu*I and *Bgl*II site) for p53-binding sites of HSPB7 were: region 1 forward, 5′-AAAACGCGTTCCAAGGTCACACAGCAGAG-3′; and reverse, 5′-TTTAGATCTGCTTCAAACCGGTCATCCT-3′; and region 2 forward, 5′-AAAACGCGTTGAGCAGGAGCA GTCAGAGA-3′; and reverse, 5′-TTTAGATCTAGCCCCAAG AGGACAAAGTT-3′.

H1299 cells were seeded in 12-well plates (5×10^4^ cells per well). Twenty-four hours later, cells were co-transfected with i) 25 ng of the pRL-CMV vector (Promega) (for internal control); ii) 125 ng of either pcDNA3.1(+)-wild-type p53 or pcDNA3.1(+) empty vector; and iii) 125 ng of pGL3-promoter vector with either the p21 promoter region corresponding to p53-binding site (for positive control) (20), that with p53-binding site 1 of HSPB7, that with p53-binding site 2 of HSPB7, or pGL3-Promoter mock vector (for negative control) by using FuGENE 6 transfection reagent (Roche). After 36-h incubation, luciferase activity was measured using the Dual Luciferase Assay System (Promega) (21).

### Statistical analysis

All statistical analyses including t-test and Fisher’s exact test were carried out by using the SPSS software (version 17). Data are shown as mean ± SD. All tests were 2-sided and p-value of <0.05 was considered to indicate a statistically significant difference.

## Results

### Downregulation of HSPB7 in RCC

**Figure 1.**
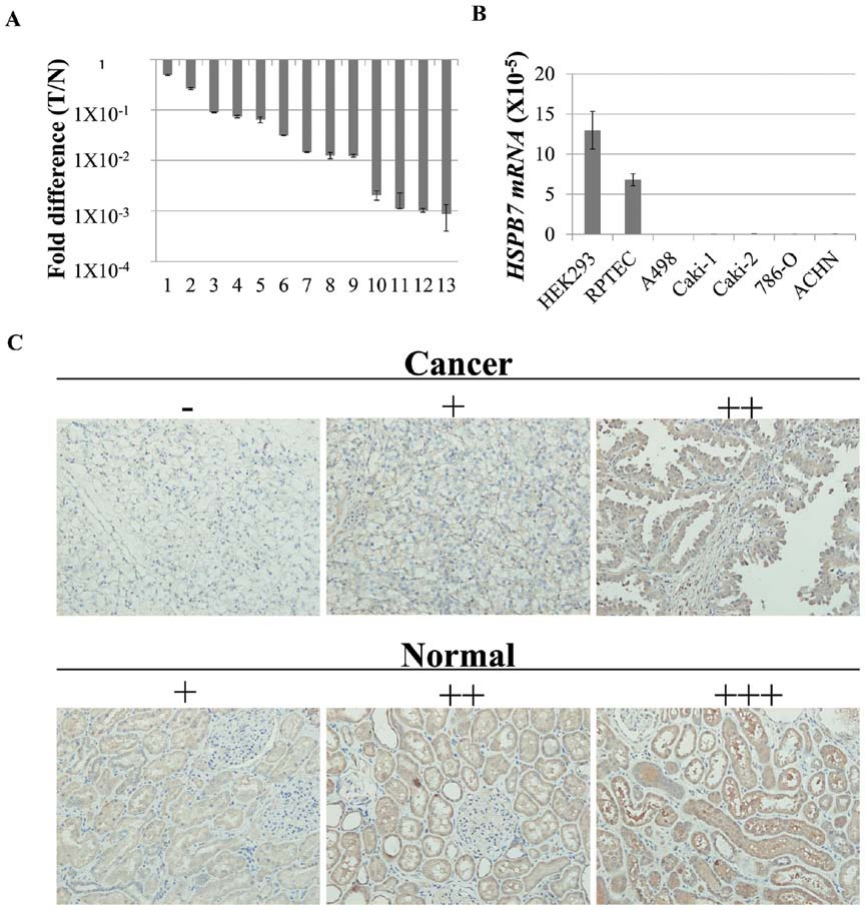
Downregulation of HSPB7 in RCC. qPCR analysis shows that HSPB7 mRNA expression was significantly downregulated (A) in 11 (85%) of 13 RCC tissues compared with the normal renal tissue, and (B) in all the five RCC cell lines compared with normal HEK 293 and RPTEC cells. T and N represent RCC tissue sample and normal renal tissue, respectively. B2M (β2 microglobulin) was used for normalization of expression levels. Values are expressed as the mean ± SD. (C) IHC analysis of a tissue array consisting of 11 pairs of human RCC sample reveals that the expression of HSPB7 was significantly higher in normal kidney tissues than in RCC tissues. According to the intensity of HSPB7 staining, these samples were evaluated as: negative (−), weakly positive (+), moderate positive (++), and strong positive (+++). HSPB7 negative or weakly positive (−/+) were considered low expression, and moderate or strong positive were considered high expression (++/+++). Summary of the IHC results is shown in Table I.

**Figure 2.**
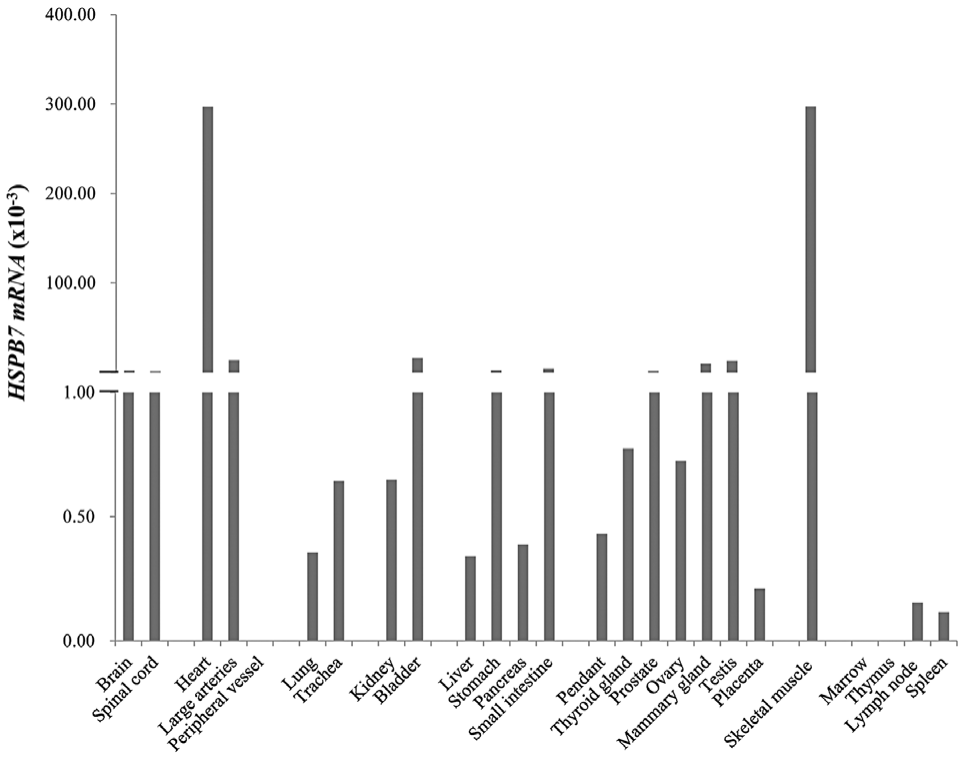
HSPB7 expression levels in normal tissues. qPCR analysis of HSPB7 was performed using mRNA isolated from 25 different normal tissues. B2M was used for normalization of expression levels.

Based on the analysis of microarray data of 15 clear cell renal cell carcinomas, we found HSPB7 to be significantly and commonly downregulated in RCC. qPCR experiment confirmed its downregulation in 11 (85%) of 13 RCC tissues and in all of the five RCC cell lines (Fig. 1A and B), compared with their corresponding normal controls. IHC analysis of a tissue array consisting of 11 pairs of human RCC sample revealed that the expression of HSPB7 was significantly higher in normal kidney tissues than that in RCC tissues (Fig. 1C and Table I). We also detected HSPB7 expression mainly in the cytoplasm of normal renal tubular epithelial cells. To explore the expression patterns of HSPB7 in other normal tissues, we performed qPCR analysis using mRNAs isolated from 25 normal tissues. HSPB7 expression was detected ubiquitously in human tissues (Fig. 2).

### 5-Aza-dC treatment restores HSPB7 expression in RCC cell lines

**Figure 3.**
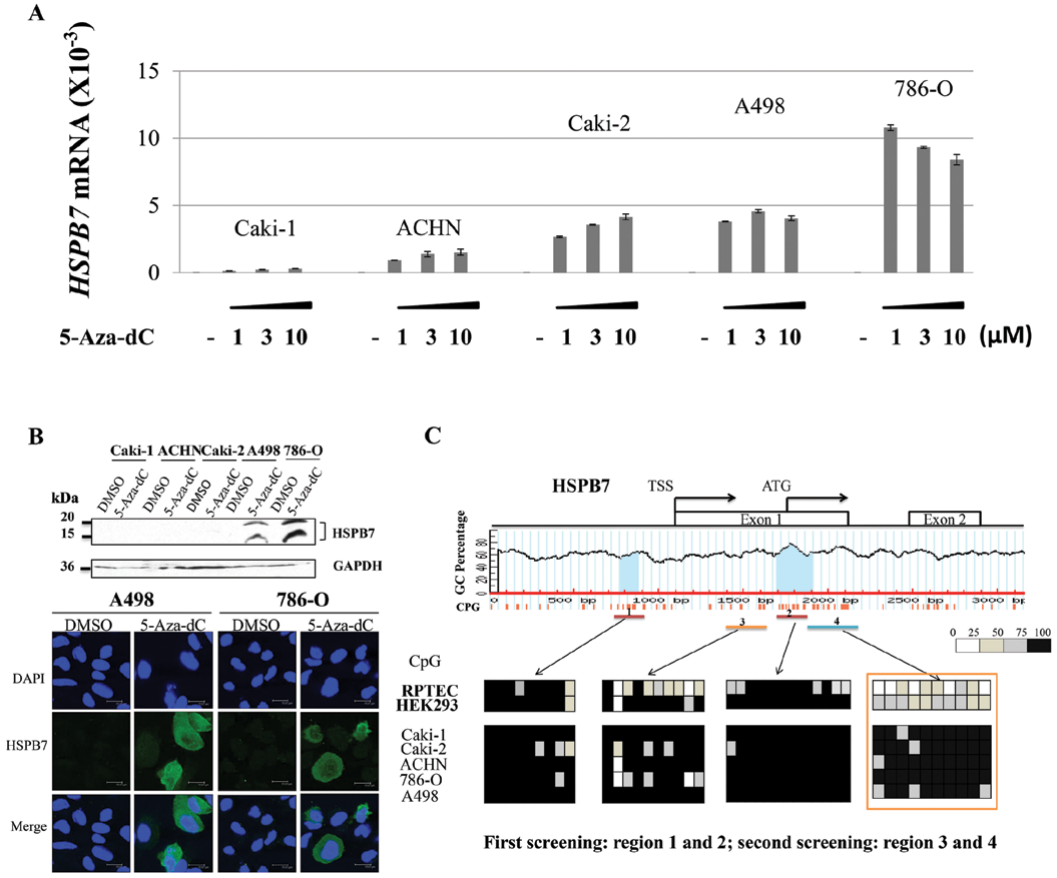
Epigenetic silencing of HSPB7 in RCC cell lines. (A) qPCR analysis and (B) western blot and ICC analysis of HSPB7 were performed in five RCC cell lines with treatment of the demethylating agent 5-Aza-dC. B2M was used for normalization of mRNA expression levels. GAPDH was used for normalization of protein expression levels. Values are expressed as the mean ± SD. (C) Hypermethylation of HSPB7 was confirmed by means of bisulfite sequencing. For each of the regions 1–4 in the cell lines, 10 or more colonies were randomly chosen and sequenced. Each square indicates a CpG site, and an average methylation level per CpG site is indicated by % methylation (shown in different color): white, 0–25% methylation; bright grey, 26–50% methylation; dark grey, 51–75% methylation; and black, 76–100% methylation. Region 4 showed higher level of methylation in the five RCC cell lines (Caki-1, Caki-2, ACHN, 786-O and A498) than in the two control cell lines (RPTEC and HEK293).

To investigate whether the methylation status of the HSPB7 gene could affect HSPB7 expression in RCCs, 5 RCC cell lines, Caki-1, Caki-2, ACHN, 786-O and A498 were treated with a demethylating agent 5-Aza-dC, and then the expression levels of HSPB7 were analyzed by qPCR, western blot and IHC analysis. We found that HSPB7 mRNA expression were restored in all the 5 RCC cell lines by the treatment with 5-Aza-dC (Fig. 3A), and the HSPB7 protein expression could also be detected in two cell lines, 786-O and A498, in which mRNA expression was most highly induced (Fig. 3B), indicating suppression of HSPB7 in RCC was caused probably by DNA hypermethylation. We performed exon sequencing of HSPB7 in these five RCC cell lines, but no mutation or deletion/insertion was detected (data not shown).

### Hypermethylation of HSPB7 in RCC

To confirm the methylation status of the HSPB7 gene, bisulfite sequencing was performed for the 5 RCC cell lines Caki-1, Caki-2, ACHN, 786-O and A498 as well as 2 normal renal cell lines RPTEC and HEK293. We first screened two CpG islands, regions 1 and 2 shown in Fig. 3C, but no significant difference of methylation status was found in these two regions in normal and cancer cell lines. Then, we performed the second screening for regions 3 and 4 (Fig. 3C) (we also screened the other regions in normal cells, but data are not shown). In region 4, we found significantly higher levels of methylation in the 5 RCC cell lines than in the 2 normal renal cell lines.

### Ectopic HSPB7 expression suppresses RCC cell clonogenicity

**Figure 4.**
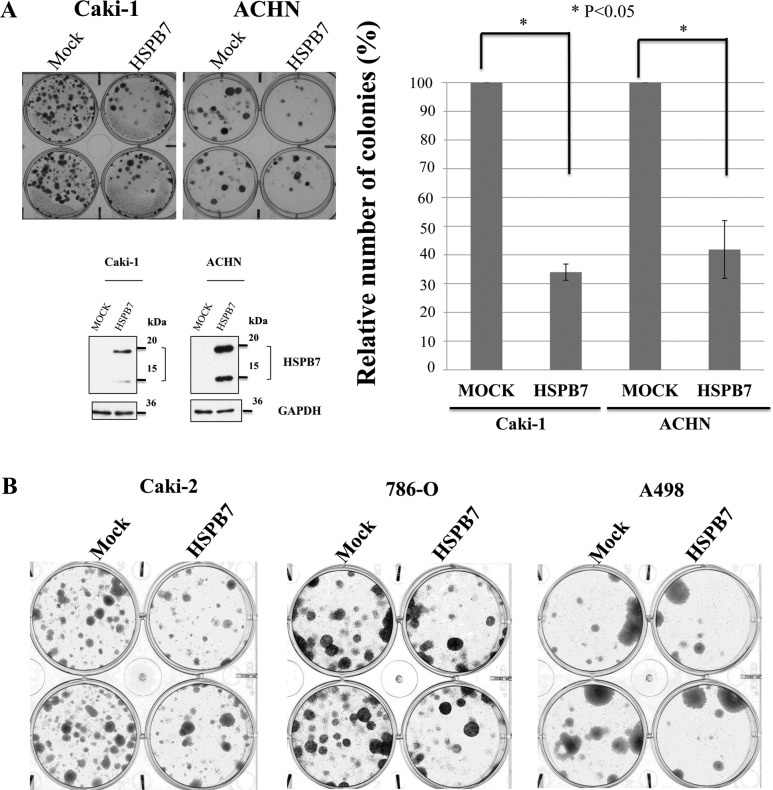
Ectopic HSPB7 expression suppresses RCC cell growth. (A) Colony formation assay showed that introduction of HSPB7 impaired colony-forming ability of Caki-1 and ACHN cells. Cells were transfected with plasmid expressing HSPB7 or mock plasmid, and colonies (>1 mm diameter) were counted after selection of 2–3 weeks with G418. At 48 h after transfection, total protein of cells was collected and applied for western blot to confirm the successful transfection. GAPDH was used for the normalization of protein expression levels. (B) Colony formation assay in Caki-2, 786-O and A498 RCC cell lines. Values are expressed as the mean ± SD.

To study the effect of HSPB7 expression on tumor growth, Caki-1 and ACHN cells were transfected with HSPB7 expression vector, pCAGGSnHC-HSPB7-HA. Introduction of HSPB7 into these two cancer cell lines caused significant decrease in the number of colonies, compared with corresponding mock-transfected controls (Fig. 4A). We also performed colony formation assay in 3 other RCC cell lines (Caki-2, A498 and 786-O) using the same vectors, and confirmed similar growth-suppressive effects (Fig. 4B), implying that HSPB7 may function as a tumor suppressor gene.

### HSPB7 is regulated by p53

**Figure 5.**
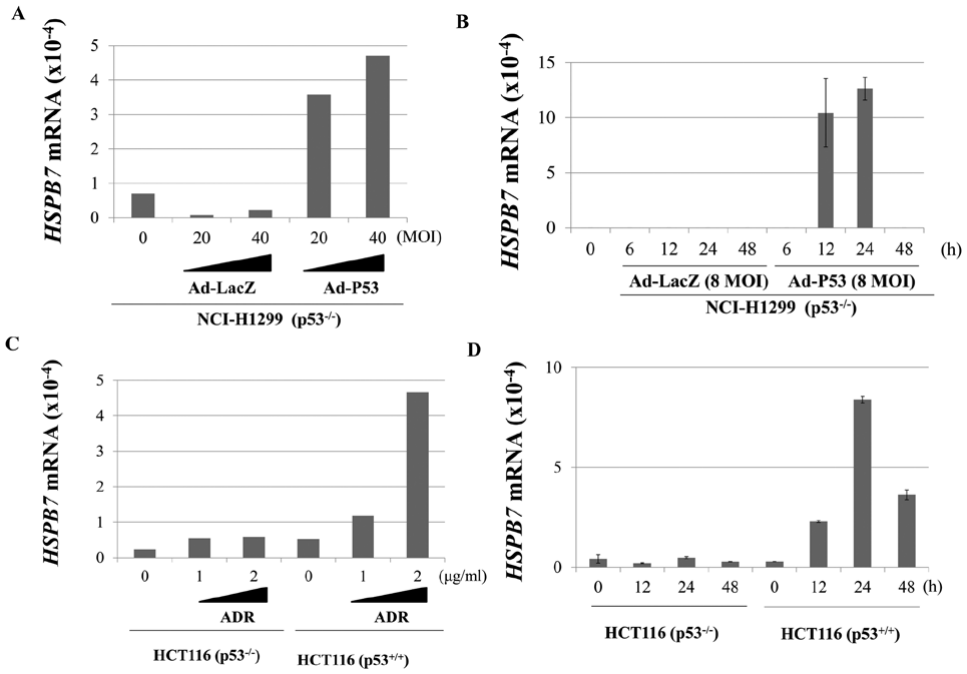
HSPB7 is regulated by p53. (A and B) HSPB7 expression in NCI-H1299 cells with or without p53 induction (A) dose- and (B) time-dependently. Cells were infected with replication-deficient recombinant adenovirus encoding p53 (Ad-p53) or LacZ (Ad-LacZ) at indicated doses, and the cells were collected 48 h later and qPCR analysis was performed (A). The cells were infected at 8 MOI and collected at different time points (B). (C and D) HSPB7 expression in HCT116 (p53^−/−^) and HCT116 (p53^+/+^) cells treated with adriamycin at indicated doses for 2 h and the cells were harvested at 48 h (C). The cells were treated with adriamycin at 2 *μ*g/ml for 2 h and then harvested at different time points (D). B2M was used for normalization of expression levels. Values are expressed as the mean ± SD.

To further elucidate the biological significance, we first investigated its possible involvement in the p53-pathway because α B-crystallin, one of the small heat shock protein family members, was reported to be induced by p53 (22,23). We applied qPCR analysis to evaluate the expression of HSPB7 in NCI-H1299 (p53 null) cell lines with or without introduction of p53 using the adenovirus system. After the infection of Ad-p53, we observed induction of HSPB7 in a dose- and time-dependent manner (Fig. 5A and B), while no induction was observed in the control cells. After the 48-hour treatment with 40 MOI of Ad-p53, the expression level of HSPB7 became nearly 5 times higher than the control cells (Fig. 5A). Induction of HSPB7 was also confirmed under the treatment with relative lower dose of Ad-p53 (8 MOI) at different time points. Concordantly, DNA damage by adriamycin treatment induced HSPB7 expression in HCT116 cells with wild-type p53, but not in HCT116 cells without wild-type p53 (Fig. 5C and D), indicating that HSPB7 expression is regulated by wild-type p53. To further investigate whether HSPB7 is directly regulated by p53, we screened two possible p53-binding sites indicated by the p53-binding site search software developed by us, but neither of these two candidate sites was confirmed to be a direct p53-binding site (data not shown). Although there might be another site(s) that p53 binds to, we are unable to conclude whether HSPB7 is directly or indirectly regulated by p53, it is certain that HSPB7 expression is inducible by wild-type p53.

## Discussion

Scarce knownledge exists on the biological function of HSPB7, a member of the small heat shock protein family that is characterized by possessing a conserved α-crystallin domain. HSPB7 has been shown to interact with the cytoskeletal protein α-filamin (24) as well as other small heat shock proteins (25). HSPB7 belongs to a non-canonical HSPB protein that prevents the aggregation of polyQ proteins in an active autophagy machinery, but overexpression of HSPB7 alone did not affect the autophagy event (26). Several genome-wide association studies found that SNPs in the HSPB7 gene were strongly associated with idiopathic cardiomyopathies and heart failure (27–31). Recently, HSPB7 was suggested to regulate early developmental steps in cardiac morphogenesis (32). However, the involvement of HSPB7 in carcinogenesis has not been described.

Through the genome-wide expression analysis in RCCs, we identified HSPB7 as a candidate tumor suppressor gene because of its common and significant downregulation in RCCs. Subsequent functional analysis revealed that HSPB7 was downregulated in cancer cells by hypermethylation. Bisulfite sequencing of genomic regions of HSPB7 confirmed hypermethylation in RCC cell lines. Although region 4 (Fig. 3C) contained no CpG Island, we observed significantly higher level of methylation in RCC cell lines than normal cell lines. Consistently, restoration of HSPB7 expression was observed by the treatment of cancer cells with 5-Aza-dC. In addition, since no somatic changes in coding regions of the HSPB7 gene were found in our sequence analysis of RCC cell lines or in the COSMIC database, HSPB7 in RCC is considered to be downregulated mostly by hypermethylation.

The second key finding in this study is that HSPB7 showed growth suppressive effect in cancer cells. Ectopic expression of HSPB7 significantly impaired colony-forming ability for 5 RCC cell lines, indicating that HSPB7 may function as a tumor suppressor gene. Similarly α B-crystallin, one of the small heat shock protein family members, was also indicated to function as a tumor suppressor in nasopharyngeal carcinoma cells (33). Furthermore, the region on chromosome 1p36.23-p34.3, where HSPB7 is located, showed frequent loss of heterozygosity in many types of solid tumors (34). However, further studies are needed to clarify the detailed tumor suppressor function of HSPB7 in RCC.

The third important finding in this study is that HSPB7 was likely to be involved in the p53 pathway. The expression of HSPB7 was significantly induced in p53-dependent manner that was clearly demonstrated by two experiments, i) that introduction of adeno-p53 in p53-negative cancer cells showed strong induction of HSPB7 and ii) that DNA-damage-dependent introduction of HSPB7 was observed in HCT116 cells with wild-type p53, but not in those lacking p53. Although we failed to identify the p53-binding site in or near the HSPB7 gene, these two pieces of evidence strongly imply a critical role of HSPB7 as the direct/indirect p53-signal transducer and its downregulation may be involved in the development of various types of cancer including RCC.

In conclusion, we carried out a genome-wide gene expression analysis and identified HSPB7 to be a candidate tumor suppressor gene in RCC. We confirmed downregulation of this gene caused by DNA hypermethylation, its growth suppressive effect in RCC cell lines and its p53-dependent expression, indicating the important roles of HSPB7 in renal carcinogenesis. Our finding could contribute to better understanding of the novel function of HSPB7 in cancer.

## Figures and Tables

**Table I.** Immunohistochemical expression of HSPB7 in RCC tissue array.

	Total	Low (−/+)	High (++/+++)	Fisher’s t-test
Clear cell				
Cancer	9	7	2	P=0.015
Normal	9	1	8	
Papillary				
Cancer	2	1	1	-
Normal	2	0	2	
Total				
Cancer	11	8	3	P=0.008
Normal	11	1	10	

All tests were 2 sided and P<0.05 was considered to indicate a statistically significant difference.
